# Kidney organoids as a novel platform to evaluate heat‐stress‐induced acute kidney injury pathogenesis

**DOI:** 10.1002/btm2.70092

**Published:** 2025-11-20

**Authors:** Qisheng Su, Liang Yue, Leixing Ge, Meida Xiang, Qi Liu, Jiru Wang, Zhimin Yun, He Liu, Congji Shan, Hebing Chen, Chengjun Wu, Zhuo Gao, Yingxia Tan

**Affiliations:** ^1^ Academy of Military Medical Sciences Beijing China; ^2^ Department of Clinical Laboratory First Affiliated Hospital Guangxi Medical University Nanning China; ^3^ Faculty of Medicine Dalian University of Technology Dalian China; ^4^ School of Health and Life Sciences Qingdao Central Hospital, University of Health and Rehabilitation Sciences Qingdao China; ^5^ Department of Nephrology Air Force General Hospital Beijing China

**Keywords:** acute kidney injury, biomarkers, heat stress, kidney organoids, nascent and steady‐state RNA sequencing

## Abstract

Acute kidney injury (AKI) is a serious condition with significant global impact. To explore mechanisms and biomarkers of heat‐stress‐induced AKI, we used human kidney organoids derived from induced pluripotent stem cells via suspension culture. Organoids were exposed to 37, 39, and 41°C. At 41°C, we found the viability decreased over time, with cytoskeleton damage, impaired tubule absorption, apoptosis, and collagen deposition. Under extreme heat (41°C), elevated AKI markers KIM‐1 and NGAL, along with cell cycle arrest markers TIMP‐2*IGFBP7 were detected. Notably, TIMP‐2*IGFBP7 appeared at 12 h post‐exposure, preceding NGAL and KIM‐1. Nascent and steady‐state RNA analyses revealed suppressed oxidative phosphorylation and ATP metabolism, along with elevated histone expression, implicating their roles in heat‐induced AKI. The data support that kidney organoids serve as a valuable model for investigating heat‐induced AKI, providing insights into early injury biomarkers that are valuable for the development of treatments.

**Translational Impact Statement**

This study establishes human iPSC‐derived kidney organoids as a high‐fidelity, human‐relevant model for heat‐stress‐induced acute kidney injury (AKI). We identified TIMP‐2*IGFBP7 as potential early urinary biomarkers for heat‐induced AKI, preceding the rise of NGAL and KIM‐1. Furthermore, our nascent and steady‐state RNA sequencing revealed suppressed oxidative phosphorylation and a metabolic shift towards glycolysis as key pathological mechanisms, alongside the novel finding of transcriptional upregulation of histones. These insights provide a foundation for developing early diagnostic strategies and targeted therapeutic interventions for populations at risk of heat‐related kidney damage, such as military personnel, outdoor laborers, and individuals in heat‐vulnerable regions, ultimately aiming to reduce the global burden of AKI.

## 1 INTRODUCTION

Heat waves are one of the top 10 global causes of death by natural disasters from 1980 to 2017, ranking 7^th^.^1^ In the past 50 years, the worldwide average temperature has increased by 0.8–0.9°C, exacerbating the frequent occurrence of heat extremes.^2^ The kidney plays a crucial role in the body's adaptation to high‐temperature environments, regulating electrolyte balance and preventing dehydration.^3^, ^4^ Prolonged exposure to high temperatures can result in heat stroke, with hyperthermia (typically defined as a temperature >40.6°C) and acute kidney injury (AKI) being common complications.^5^, ^6^ AKI is characterized by a rapid decline in renal function in the short term. The diagnosis of AKI is based on serum creatinine levels and urine output according to KDIGO (Kidney Disease: Improving Global Outcomes) criteria.^7^, ^8^ Currently, there are no effective, specific treatments for AKI. The “0by25” initiative seeks to eradicate all preventable deaths associated with AKI by the year 2025.^9^ Therefore, further research is needed to understand the mechanisms of heat‐induced AKI, identify early diagnostic markers, and develop effective treatments for affected individuals.^10^

At present, the primary experimental models used for studying AKI are animal models and two‐dimensional (2D) cell lines.^11^, ^12^ However, the animal model is limited by species differences and ethical concerns. On the other hand, a 2D cell model can only provide a single type of renal cell and has lower levels of transport proteins and metabolic enzymes compared to in vivo normal physiological conditions, making it challenging to accurately recapitulate the complex biological environment.^13^, ^14^ In recent years, kidney organoids derived from human iPSCs are becoming attractive and widely used in disease modeling, such as drug nephrotoxicity testing, gene‐deficient disease, inflammatory kidney disease, and viral infectious disease.^15^, ^16^, ^17^ Several differentiation protocols have been established to induce kidney organoids.^18^, ^19^, ^20^

In this study, we generated 3D kidney organoids from human iPSCs, recapitulating key features of the native kidney, including glomeruli, segmented tubules, interstitial cells, and endothelial cells, as previously described.^21^ Using this organoid, we established an in vitro model of heat‐stress‐induced AKI to investigate the pathological characteristics and potential mechanisms involved in this condition. The findings revealed that heat‐stress‐induced AKI exhibited structural and absorptive damage, accompanied by increased collagen fiber deposition. The injury biomarkers, such as tissue inhibitor of metalloproteinases‐2 * ﻿﻿insulin‐like growth factor‐binding protein 7 (TIMP‐2*IGFBP7), were identified as potential early predictive indicators of AKI. Additionally, analysis of nascent RNA and steady‐state RNA demonstrated downregulation of oxidative phosphorylation and ATP metabolism, along with upregulation of histone expression, potentially contributing to the pathology of heat‐stress‐induced AKI. This research represents a pioneering exploration of the mechanisms and pathological alterations associated with heat‐stress‐induced AKI using kidney organoids.

## 2 METHODS

### 2.1 Generation of human kidney organoids

The research‐grade human‐induced pluripotent stem cell line used in this study was purchased from Nuwacell Biotechnologies Co., Ltd. (RC01001‐A). The protocol for generating kidney organoids was referred to as the method described by Przepiorski et al.^21^ Human iPSCs were gradually differentiated through the stages of embryoid bodies (EBs), intermediate mesoderm (IM), metanephric mesenchyme (MM), and ureteric bud (UB). The sequential process led to the generation of renal organoids that comprised nephron units, mesenchymal tissue, and vascular endothelial tissue.

The composition of the culture medium is outlined in Table S1, Supporting Information. When reached 70% confluence, iPSCs were detached using and incubated until the cells were fully digested. The cell suspension was then centrifuged to remove the supernatant, and the cell pellet was resuspended in Day 0 medium to achieve a concentration of 4000 cells per well. This cell suspension was then dispensed into a 96‐well U‐bottom plate at 100 μL per well using a multichannel pipette. The plate was sealed, centrifuged at 200*g* for 6 min, and incubated at 37°C with 5% CO_2_ for 48 h to allow for cell aggregation. By Day 2, EBs were visible at the well bottom, which were subsequently transferred to a 6‐well plate, resuspended in 2 mL of Day 2 medium, and placed on a shaker at 70 rpm for 24 h. On Day 3, the medium was changed to Day 3 medium, and the cells were cultured until maturity, with medium renewal every 2 days. The maturity of the kidney organoids was evaluated by analyzing their transcriptional profiles for the presence of key developmental markers characteristic of human fetal kidney tissues, as previously described.^12^, ^22^ This included markers for anterior intermediate mesoderm (e.g., LHX1, GATA3), posterior intermediate mesoderm (e.g., HOXD11, EYA1, TBX6), and segment‐specific nephron structures (e.g., UMOD, NPHS1, CUBN).

### 2.2 Dextran absorption experiment

A 10‐kDa rhodamine‐dextran (Invitrogen, D1824) was dissolved in Day 3 medium at a concentration of 20 mg/mL and incubated with kidney organoids for 6 h. Following incubation, the organoids were thoroughly washed with DPBS 3–5 times and then fixed with 4% paraformaldehyde (PFA) overnight at room temperature. Subsequently, the fixed organoids were embedded in paraffin and then sectioned into 4 μm slices. Standard fluorescence staining protocols were followed, with DAPI used for nuclear staining. Imaging of the organoid sections was carried out using a PerkinElmer Vectra 3 System.

### 2.3 Proliferation and viability of kidney organoids

The proliferation and viability of renal organoids were evaluated using the LIVE/DEAD™ Viability/Cytotoxicity Kit (Thermo Fisher, L3224) and ATP measurement (CellTiter‐Glo 3D Cell Viability Assay, Promega, G9681). For the Live/Dead assay, live cells were stained green, while necrotic cells showed red fluorescence, assessed using a Nikon A1 confocal microscope. The number of viable cells was also determined based on the presence and quantity of ATP. One‐hundred microliter of CellTiter‐Glo 3D reagent was added to each well with 100 μL of culture medium containing three organoids. After incubating for 25 min at room temperature, bioluminescence activity was measured using a plate luminometer. Viability was monitored at various time points (0, 3, 6, 9, 12, 24, 36 h) during heat treatment.

### 2.4 Histological evaluation of kidney organoids

After 14 days of culture, the organoids were fixed in 4% PFA. Paraffin embedding and sectioning were performed as described above. Deparaffinization and hematoxylin and eosin (H&E) staining were conducted according to standard protocols. Imaging of the organoid sections was performed using a PerkinElmer Vectra 3 System.

### 2.5 Masson staining

Deparaffinization was conducted according to standard protocol. Subsequently, the sections were stained with Weigert's hematoxylin for 5–10 min to visualize the nuclei, followed by rinsing with running water. They were then stained with an acid fuchsin solution for 5–10 min to stain the cytoplasm and muscle fibers, followed by gentle rinsing to remove excess dye. The sections were differentiated in a phosphomolybdic acid solution for 5–10 min to enhance connective tissue visibility and stained with an aniline blue or light green solution for 5–10 min to stain the collagen fibers of the connective tissue. After staining, the sections were dehydrated in 95% ethanol, cleared with xylene, and finally mounted with neutral resin and dried.

### 2.6 Immunofluorescence

The Organoids were fixed in 4% PFA, followed by paraffin embedding and sectioning as described above. Immunofluorescence (IF) was conducted using standard procedures, which included heat‐induced antigen retrieval and incubation in a protein blocking buffer. DAPI was used for nuclear staining. Specific primary anti‐human antibodies used were: LTL (Invitrogen™, L32480), PECAM1/CD31 (abcam, ab76533), nephrin/NPHS1‐B‐12 (Santa Cruz, sc‐377246), E‐Cadherin/ECAD/CDH1 (abcam, ab231303), MEIS1/2/3(Santa Cruz, sc‐101850), SLC12A1 /NKCC2 (proteintech, 18970‐1‐AP), Integrinα6 (Santa Cruz, sc‐13542), and F‐actin/NH3 (invitrogen, MA1‐80729). After incubation with the primary antibody overnight at 4°C, the corresponding diluted secondary antibodies were applied to the slides for 1 h in the dark at room temperature. All slides were imaged using PerkinElmerVectra3 System.

### 2.7 Immunohistochemistry

Immunohistochemistry was performed according to standard procedures. Following deparaffinization and rehydration, antigen retrieval was carried out with citrate buffer (pH 6.0) for epitope exposure. Sections were incubated with 3% hydrogen peroxide to block endogenous peroxidase activity, followed by a protein blocking buffer to reduce nonspecific binding. Then, slides were incubated overnight at 4°C with anti‐α‐SMA (Abcam, ab7817) for myofibroblast detection. After washing, HRP‐labeled secondary antibodies were applied for 1 h at room temperature. Visualization was achieved using a DAB substrate, which produced a brown precipitate at the antigen sites. Finally, sections were counterstained with hematoxylin, dehydrated, cleared, and mounted. Protein expression and localization were evaluated using a bright‐field microscope.

### 2.8 RNA extraction and quantitative real‐time PCR

Total RNAs were extracted using TRIzol reagent (Invitrogen, 155969‐026) and cDNAs were synthesized with qScript‐cDNA‐SuperMix (Quantabio, 95048‐025). Quantitative real‐time PCR (qRT‐PCR) was performed by TB Green Premix Ex Taq II (TakaRa, RR820). The primer sequences for the reaction are provided in Table 1.

**TABLE 1:** PCR primers for qRT‐PCR.

Gene of interest	Forward (5′–3′)	Reverse (5′–3′)
*β‐Actin*	CGTCACCAACTGGGACGACA	CTTCTCGCGGTTGGCCTTGG
*KIM‐1*	TGGCAGATTCTGTAGCTGGTT	AGAGAACATGAGCCTCTATTCCA
*NGAL*	ATCACTCTCAGGGTCTGCAC	GGCAGGGGAATGTGAGAACT
*TIMP‐2*	AAGCGGTCAGTGAGAAGGAAG	GGGGCCGTGTAGATAAACTCTAT
*IGFBP7*	CGAGCAAGGTCCTTCCATAGT	GGTGTCGGGATTCCGATGAC
*COL4A1*	GGACTACCTGGAACAAAAGGG	GCCAAGTATCTCACCTGGATCA
*COL4A2*	AAGGGCTTCATCGGAGACC	CCAGCGTCACCTTTCCACC
*COL5A1*	TACAACGAGCAGGGTATCCAG	ACTTGCCATCTGACAGGTTGA
*COL5A2*	CCGGGTCTAGCTGGTGAAAG	TCTCCTCTAGGTCCTAACGGG
*COL6A1*	AGGGCTACAAGGAACCCTGT	CACCGAGAAGACTTTGACGC
*COL6A2*	GACTCCACCGAGATCGACCA	CTTGTAGCACTCTCCGTAGGC
*COL7A1*	GGATGACTCGACCTCTGCTC	GTCCACTGTACTCTCAAGGATTG

### 2.9 Nascent and steady‐state RNA sequencing of kidney organoids

To specifically label nascent RNA, organoids were incubated with 0.5 mM 5‐ethynyluridine (EU) for 1 h, allowing its incorporation into newly synthesized RNA. After incubation, organoids were washed three times with pre‐warmed PBS to remove residual EU. Organoids were incubated in medium with 0.5M uridine for 1 h to mature RNA. Total RNA was extracted using TRIzol reagent (Invitrogen, 155969‐026) following the manufacturer's instructions, and RNA quality was assessed by Nanodrop spectrophotometry (260/280 ratio). Nascent RNA was labeled with biotin‐4PEG‐azide via click chemistry and enriched using streptavidin‐coupled beads (Invitrogen, 65002). The enriched RNA was eluted and prepared for sequencing. For steady‐state RNA sequencing, rRNA was removed from total RNA using rRNA depletion kit (Vazyme, N420‐02), followed by a reverse transcription step using a reverse transcription kit (Invitrogen 18090050, A48570). Libraries were prepared with the DNA Library Prep Kit (NEB, E6177) following standard protocols. Final libraries were quantified and assessed using Qubit and Agilent fragment analyzer before sequencing on an Illumina NovaSeq X plus platform to generate 150 bp paired‐end reads. Sequencing data were processed using FastQC for quality assessment, followed by adapter trimming and low‐quality read removal using Fastp, and clean data were used for downstream analysis.

### 2.10 Bioinformatics analysis

Clean data were mapped to hg38 using hisat2 and quantified by featureCounts. Using the limma package in R, differential expression analysis was performed on gene expression data between the experimental and control groups. Significant differentially expressed genes (DEGs) were identified based on linear model fitting and Bayesian testing, with the screening criteria set to |log2FoldChange| > 2 and FDR < 0.05. Subsequently, gene set enrichment analysis (GSEA) was conducted using the clusterProfiler package. All genes were ranked by their log2FoldChange values, and the GSEA function was used to identify significantly enriched gene sets (FDR < 0.05) in the Kyoto Encyclopedia of Genes and Genomes (KEGG), Reactome and Gene Ontology (GO) databases. The analysis results were visualized using ridge plots.

The transcription rate of RNA is defined as the amount of RNA produced per unit of time. Here, we calculate it by dividing the normalized RNA expression level obtained from nascent RNA sequencing by the treatment duration (3 h). Subsequently, the transcription rates of the heat treatment group and the control group were compared, and the logarithm of their ratio was used to measure the magnitude of the transcription rate change.

To explore the protein–protein interaction (PPI) relationships of the DEGs, the DEGs were uploaded to the STRING database (interaction threshold >0.4) to construct a PPI network. The network data were then imported into Cytoscape for visualization. Further, the MCODE plugin in Cytoscape was used to identify modules in the PPI network (parameters: Node Score Cutoff = 0.2, K‐Core = 2, Degree Cutoff = 2). The Python package mygene was used for the annotation of genes in each module of PPI.

### 2.11 Statistical analysis

Data are presented as mean ± standard deviation (SD). Comparisons between two groups were performed using two‐tailed Student's *t* test, assuming normal distribution and equal variance, as preliminary tests supported these assumptions. Significance was set at *p* < 0.05. Statistical results were calculated using GraphPad Prism 9 software. All analyses and data visualization with sequencing of nascent and steady‐state RNAs were conducted in R (version 4.4.2) and Python (version 3.12.7).

## 3 RESULTS

### 3.1 Generation of human iPSCs‐derived kidney organoids

**FIGURE 1:**
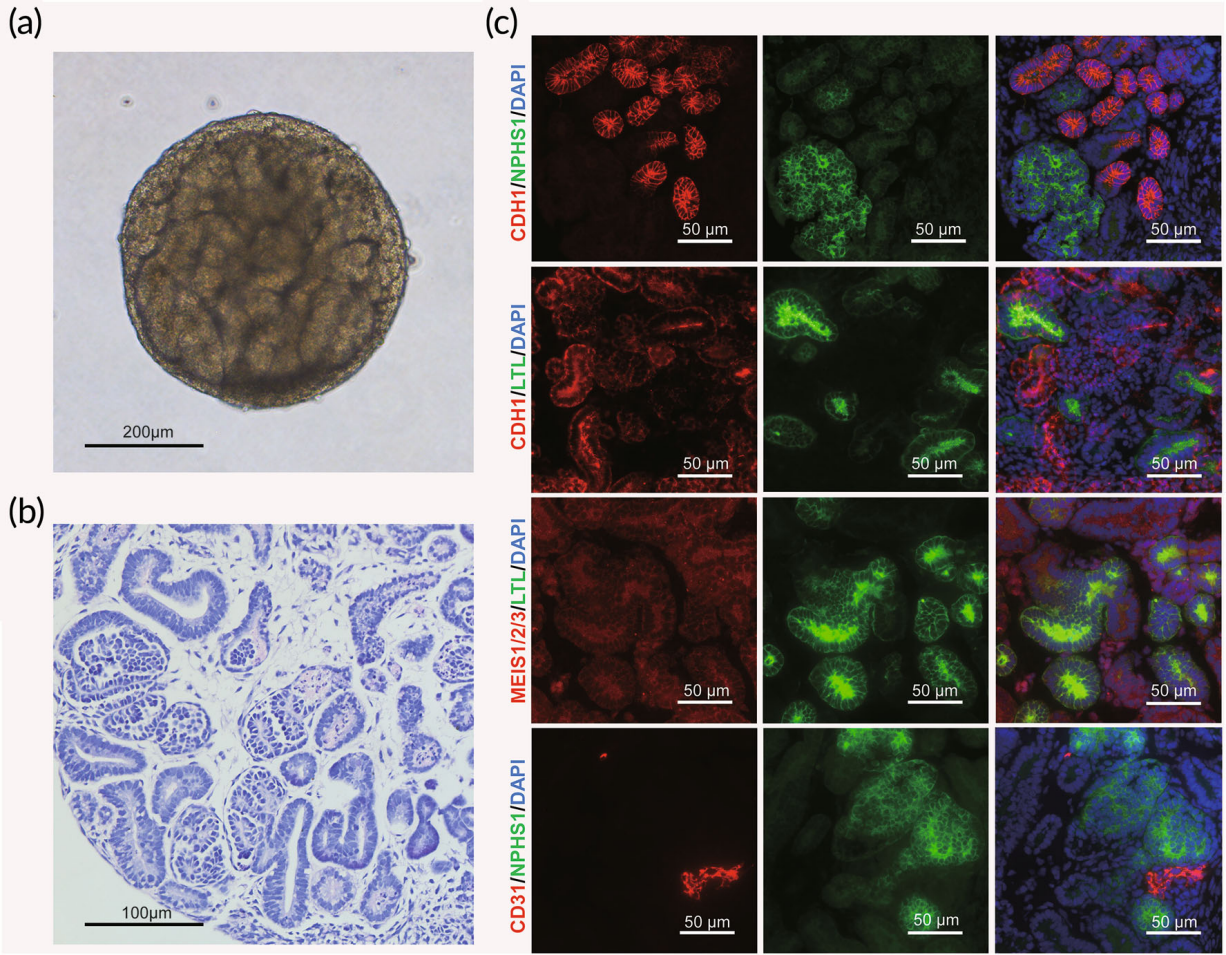
Generation of human iPSCs‐derived kidney organoids. Tubular structures were observed in the kidney organoids (a). On Day 14, H&E staining revealed the key nephron structure components such as tubules, glomeruli, and interstitium (b). Immunofluorescence (IF) identified kidney‐specific markers: Lotus tetragonolobus lectin (LTL) for proximal tubules; Epithelial cadherin (ECAD) for distal tubules; Platelet endothelial cell adhesion molecule‐1 (PECAM‐1/CD31) for vascular endothelial cells; SLC12A1 (NKCC2) for Henle's loop; Nephrin (NPHS1) for podocytes of glomeruli; and MEIS1/2/3 for stromal cells (c).

Kidney organoids were successfully established using the suspension culture method as described by Przepiorski et al.^21^ The differentiation process of human iPSCs involved sequential stages including EBs, IM, MM and UB, resulting in the formation of nephron‐like organoids. By Day 8, obvious tubular structures began to appear inside the kidney organoids (Figure 1a). HE staining on Day 14 revealed key nephron structures such as tubules, glomeruli, and interstitium (Figure 1b). IF analysis was utilized to confirm the expression of kidney‐specific markers (Figure 1c). Specifically, *Lotus tetragonolobus* lectin (LTL) labeled proximal tubules; Epithelial cadherin (ECAD) labeled distal tubules; Platelet endothelial cell adhesion molecule‐1 (PECAM‐1/CD31) was used to label vascular endothelial cells; SLC12A1 (also known as NKCC2) marked the Henle's loop; Nephrin (NPHS1) labeled the podocytes of the glomerulus; MEIS1/2/3 labeled the stromal cells. The results indicated that we have successfully constructed kidney organoids consisting of proximal tubular and distal tubular epithelium, Henle's loop, vascular endothelial cells, podocytes, and renal interstitial cells. To evaluate the maturity of the kidney organoids, transcriptional profiles were analyzed to identify key developmental markers characteristic of human fetal kidney tissues, as previously described.^21^, ^22^ Our analysis revealed predominant expression of fetal‐stage markers, such as LHX1 and GATA3 for anterior intermediate mesoderm, and HOXD11, EYA1, and TBX6 for posterior intermediate mesoderm. Additionally, the presence of segment‐specific markers like UMOD, NPHS1, and CUBN further supported a fetal kidney‐like phenotype (Figure S1).

### 3.2 Establishment of heat‐stress‐induced AKI model

**FIGURE 2:**
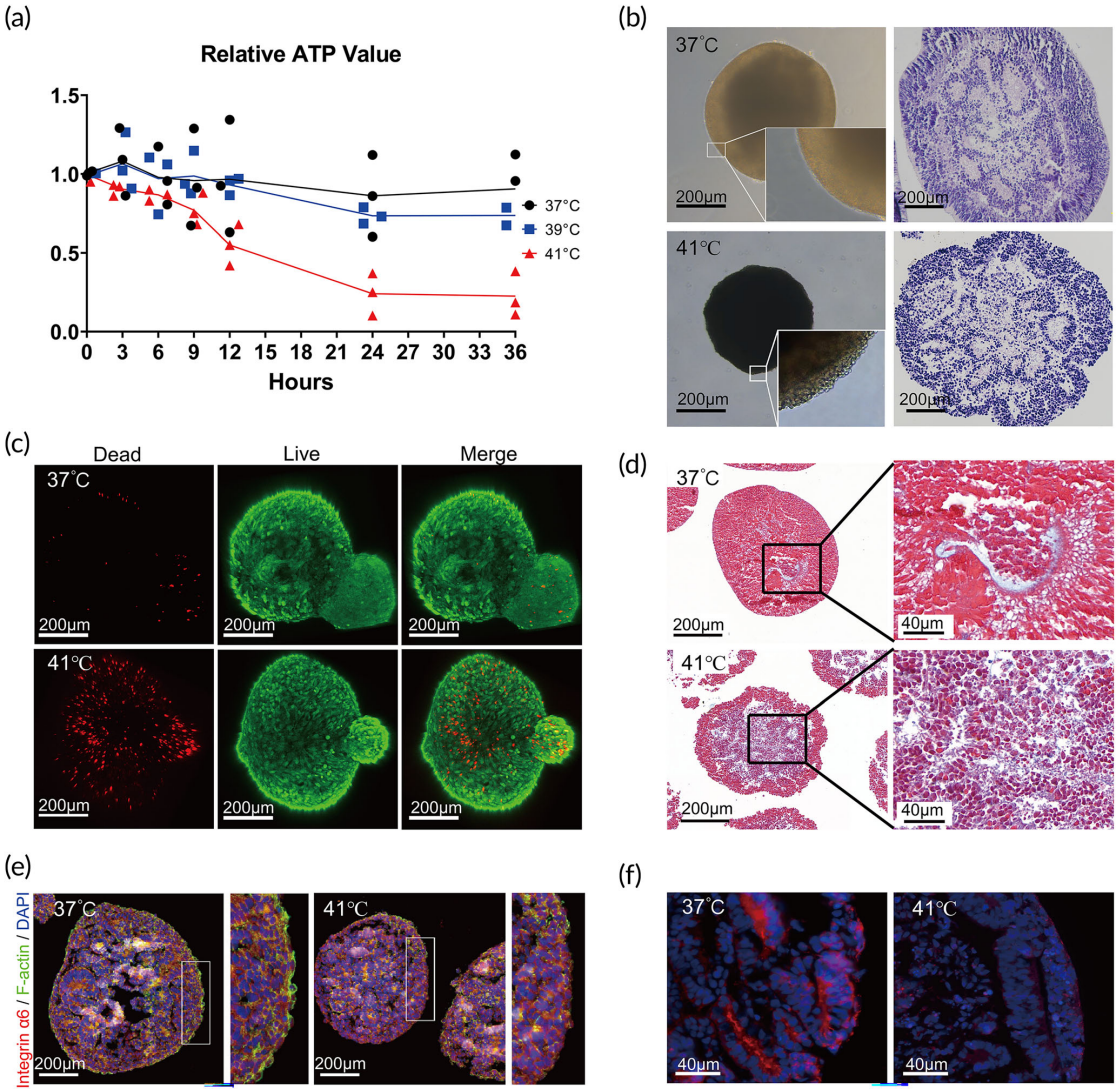
Induction of kidney organoid formation by hyperthermia. (a) Relative expression levels of ATP content compared with 0 h for each time period (*n* = 3 biological replicates). (b, c) Morphological changes of kidney organoids after heat treatment at 41°C for 24 h were observed using H&E staining and live/dead staining, revealing noticeable morphological damage, significant apoptosis, and necrosis of glomerular and tubular cells. (d) The medial fibrosis was more significant in the kidney organoid exposed to high temperature compared to the control. (e) The expression level of F‐actin significantly decreased after 24 h at 41°C, indicating cytoskeletal damage in renal organoids due to high temperature exposure. (f) The absorption function of kidney organoids was assessed using Dextran (10 kDa, Rhodamine B labeled), which appeared inside the renal tubules, indicating specific uptake by tubular cells. After 24 h at 41°C, the absorption function of renal tubules was significantly inhibited.

This study evaluated the structural integrity, viability, and absorption function of kidney organoids under different temperature conditions. Temperature gradients of 37, 39, and 41°C were set, and ATP levels were measured at intervals of 0, 3, 6, 9, 12, 24, and 36 h, respectively. The results indicated that the ATP levels in the 37°C group remained stable, whereas a significant decrease in ATP content was observed in the 39 and 41°C groups. In the 41°C group, ATP levels dropped to approximately 50% at 12 h, continuing to decline to 25% at 24 h (Figure 2a).

Subsequently, morphological and structural changes in kidney organoids were examined after 24 h of heat exposure at 41°C using HE staining and live/dead staining. Noticeable morphological damage was observed with significant apoptosis and necrosis of glomerular and tubular cells, and a reduction of tubular structures (Figure 2b,c). The degree of kidney organoid fibrosis was evaluated by Masson staining. Masson staining revealed a notable increase in blue‐stained areas (indicative of collagen fibers) after 24 h of treatment at 41°C, with a significantly larger stained area compared to the 37°C control group (Figure 2d), which indicates that the degree of medial fibrosis was significantly higher in the 41°C treatment than in the 37°C control conditions. Moreover, the expression of F‐actin significantly decreased after 24 h at 41°C, indicating cytoskeletal damage in kidney organoids (Figure 2e). In addition, the impact of high temperature on renal absorption function was assessed. Dextran (red) appeared inside the renal tubules, indicating that tubular cells of kidney organoids have absorption function However, following 24 h of high‐temperature treatment (41°C), tubular absorption function was significantly inhibited, indicating impaired tubular function (Figure 2f). Collectively, these results demonstrate that increased temperatures cause structural damage and interstitial fibrosis, as well as impair absorption function in the kidney.

### 3.3 Detection of injury biomarkers

**FIGURE 3:**
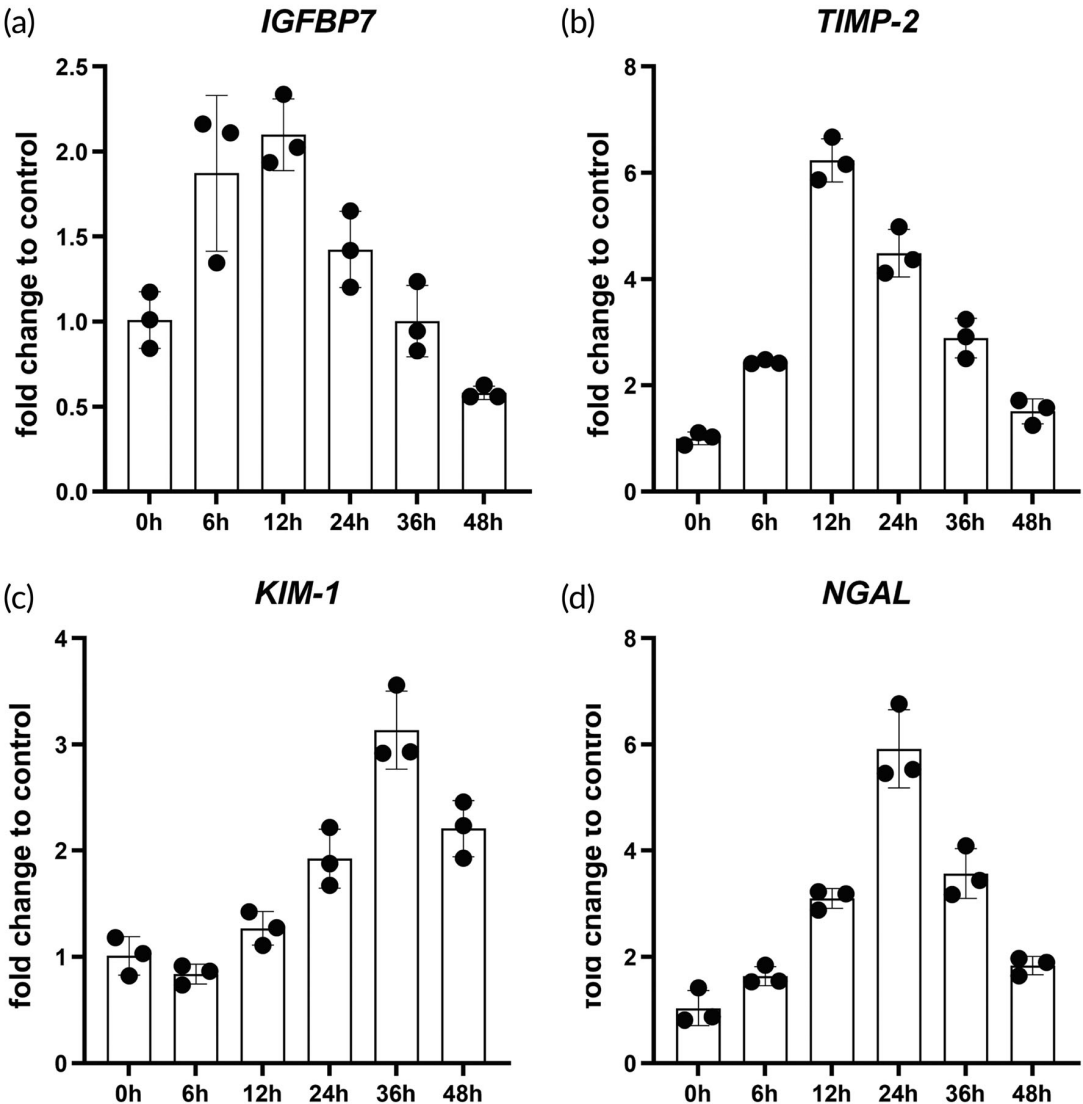
Analysis of injury biomarker expression in heat‐stressed kidney organoids. The expression of biomarkers *IGFBP7* (a), *TIMP2* (b), *NGAL* (c), and *KIM‐1* (d) in heat‐stress‐induced AKI was evaluated by qRT‐PCR at various time intervals (0, 6, 12, 24, 36, and 48 h). (*n* = 3 biological replicates).

Detecting renal injury biomarkers is crucial for diagnosing and monitoring kidney disease to track injury progression and evaluate treatment efficacy. Currently, there are early diagnostic biomarkers available to assess the risk of AKI in ICU patients, which can be detected in their urine. These injury biomarkers include TIMP‐2, IGFBP7, neutrophil gelatinase‐associated lipocalin (NGAL) and kidney injury molecule‐1 (KIM‐1).^23^, ^24^ The expression of biomarkers in heat‐stress‐induced kidney organoid injury was evaluated by qRT‐PCR at various time intervals (0, 6, 12, 24, 36, and 48 h) (Figure 3a–d). Following heat treatment, the levels of *NGAL*, *IGFBP7*, and *TIMP‐2* exhibited an initial rise, peaked, and subsequently decreased. Specifically, *IGFBP7* and *TIMP‐2* peaked at 12 h, while *NGAL* reached its peak at 24 h, and KIM‐1 at 36 h.

To further elucidate transcriptional dynamics, we performed nascent and steady‐state RNA sequencing analysis at the 24 h time point, aligning with key changes observed in the qPCR time course. Following 24 h of heat exposure at 41°C, nascent RNA analysis revealed significant upregulation of *HAVCR1* (encoding KIM‐1) and *LCN2* (encoding NGAL) in heat‐stressed organoids, whereas their steady‐state RNA levels showed no significant alterations. In contrast, steady‐state RNA levels of *TIMP2* were significantly elevated compared to controls, with no notable changes in its nascent RNA.

*IGFBP7* showed no significant differences in either nascent or steady‐state RNA levels between groups (Figure S2). Since nascent RNA reflects real‐time transcriptional activity, these findings are consistent with the qPCR results derived from total RNA, supporting a coherent pattern of biomarker response to heat stress. These early diagnostic biomarkers are consistent with the accuracy of clinical diagnosis^25^, ^26^, ^27^ and are promising for early detection of heat‐stress‐induced AKI.

### 3.4 Changes in nascent and steady‐state RNA levels after high‐temperature exposure

**FIGURE 4:**
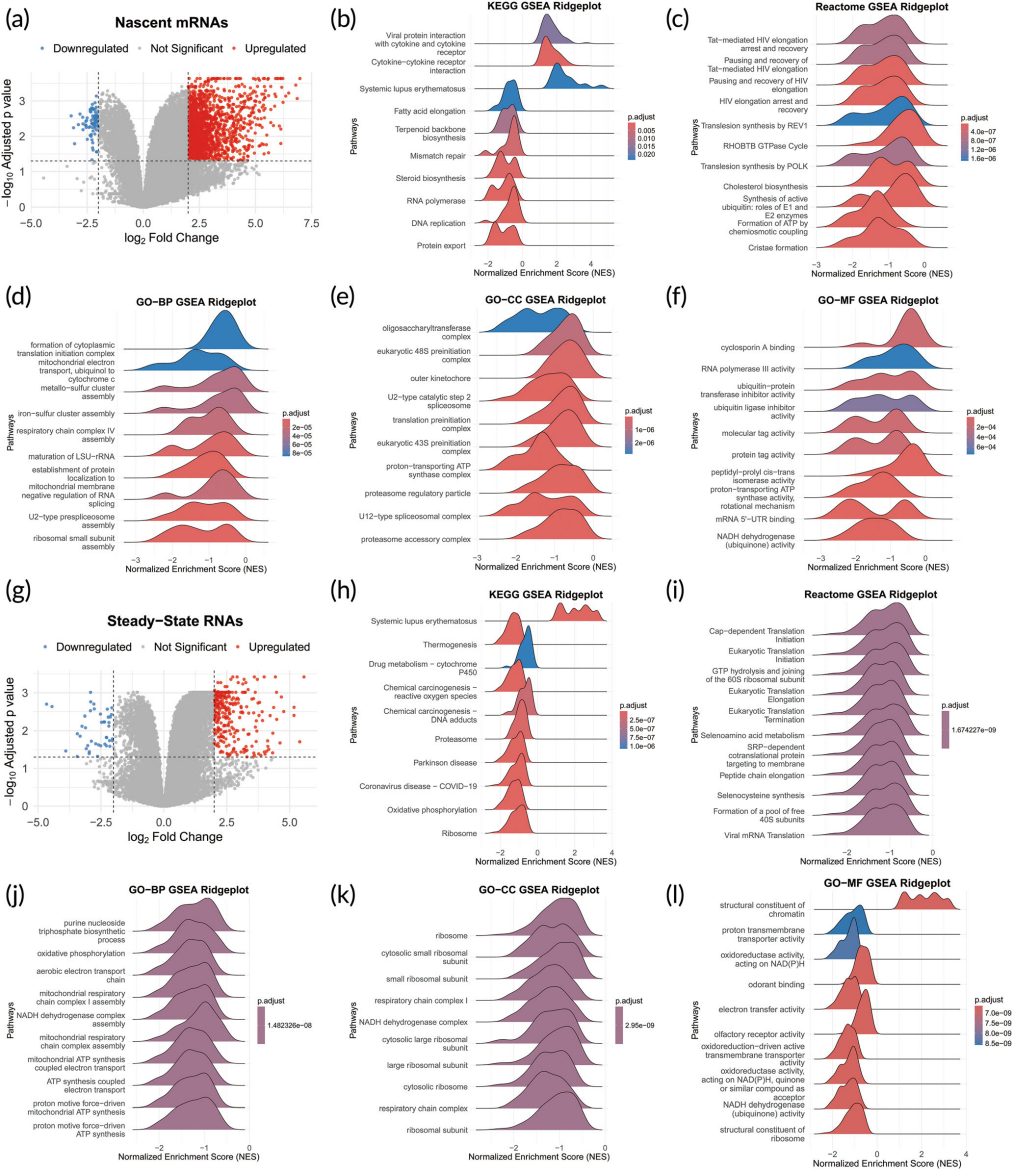
Differential Analysis and GSEA of nascent and steady‐state RNAs. Volcano plot displays differentially expressed genes in nascent RNAs (a) and in steady‐state RNAs (g). GSEA results for nascent RNAs (b–f) and steady‐state RNAs (h–l) (*n* is 3 biological replicates).

To investigate the mechanisms underlying heat‐induced AKI, we performed nascent and steady‐state RNA sequencing on kidney organoids subjected to heat stress at 41°C (heat‐induced group) and under control conditions. Analysis revealed that, in nascent RNA, 6107 genes were significantly upregulated and 822 genes were downregulated in the heat‐treated group compared to the control group (Figure 4a and Table S2). In steady‐state RNAs, 2545 genes were significantly upregulated and 466 genes were downregulated in the heat‐treated group compared to the control group (Figure 4g and Table S3). The findings indicated that nascent RNA‐seq is more sensitive in detecting transcriptional expression changes in the heat‐induced group than in the control group.

GSEA was conducted to explore the impact of heat treatment on biological processes and pathways. Notably, in nascent RNAs, genes affected by heat treatment showed significant enrichment in upregulated KEGG pathways including Protein export, RNA polymerase, and Cytokine‐cytokine receptor interaction, while downregulated GO terms encompassed U2‐type prespliceosome assembly and microRNA (miRNA)‐mediated gene silencing by mRNA destabilization, alongside Reactome pathways related to the Formation of ATP by chemiosmotic coupling (Figure 4b–f and Table S4).

In steady‐state RNAs, we observed significant downregulation of pathways and biological processes related to ATP metabolism, including oxidative phosphorylation in KEGG, respiratory electron transport in Reactome, and GO‐BP terms such as proton motive force‐driven ATP synthesis, proton motive force‐driven mitochondrial ATP synthesis, ATP synthesis coupled electron transport, mitochondrial ATP synthesis coupled electron transport, and aerobic electron transport chain (Figure 4h–l and Table S5). These findings align with our previous ATP measurements, indicating that heat treatment inhibits ATP synthesis.

### 3.5 Transcriptional rates regulate RNA expression changes after heat treatment

**FIGURE 5:**
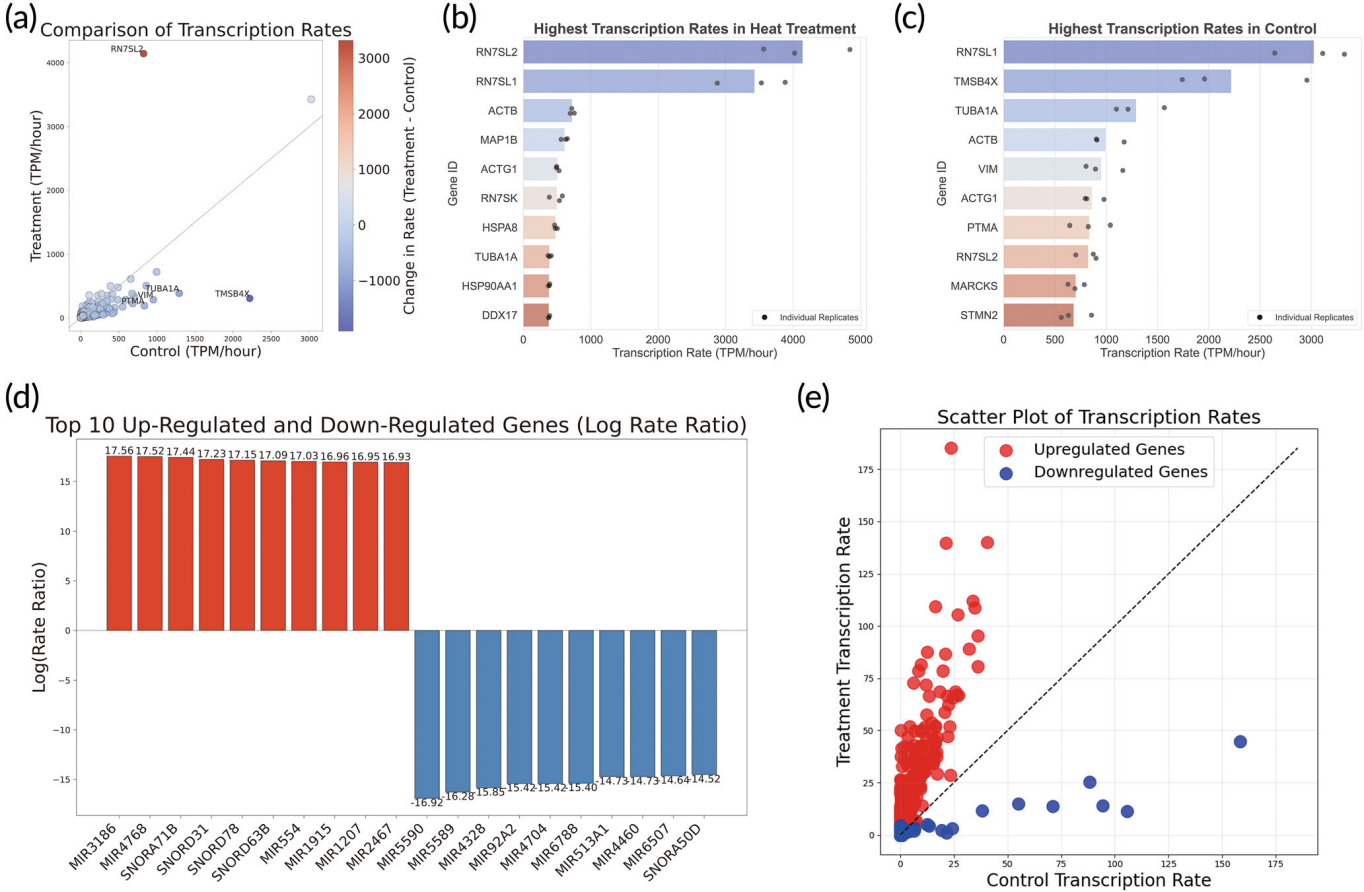
Changes in transcription rates of kidney organoids after heat treatment. (a) Comparison of transcription rates between heat treatment and control. (b, c) The top ten genes with the highest transcription rates in the heat treatment group (b) and the control group (c). (d) The top ten genes exhibiting the most notable upregulation and downregulation in transcription rates. (e) Transcription rates of differentially expressed genes in steady‐state RNAs. In the visualization: red nodes signify upregulated genes, whereas blue nodes indicate downregulated genes (*n* = 3 biological replicates).

By analyzing nascent transcriptome data, we calculated the transcription rates of the heat treatment group and the control group. It was found that the transcription rates of most genes slowed down after heat treatment (Figure 5a and Table S6). The top ten genes with the highest transcription rates in the high temperature treatment group and control group were shown in Figure 5b,c, respectively. Notably, *RN7SL2* exhibited the fastest transcription rate post high temperature treatment, ranking eighth in the control group. Genes showing the most significant changes in upregulation and downregulation (normalized using log transformation) were predominantly miRNA or small nucleolar RNA (snoRNA) encoding genes (Figure 5d). Comparison of the transcription efficiency of differentially expressed genes in steady‐state RNAs indicated increased transcription rates for upregulated genes and decreased rates for most downregulated genes (Figure 5e). These findings suggest that alterations in RNA expression post heat treatment are primarily driven by changes in transcription rates, with a minority of downregulated genes potentially linked to accelerated RNA degradation.

### 3.6 Heat treatment induces significant transcriptional changes associated with renal fibrosis in kidney organoids model

Through Masson staining analysis, we observed a significant increase in collagen accumulation in heat‐treated kidney organoids compared to the control group, indicating the induction of fibrotic changes. To further investigate the molecular mechanisms underlying fibrosis, we conducted expression analyses of collagen‐related genes, *ACTA2* (encoding α‐SMA), and two fibrosis‐related Human Phenotype Ontology terms—RENAL_FIBROSIS and RENAL_INTERSTITIAL_FIBROSIS—using steady‐state RNA expression data.^28^

**FIGURE 6:**
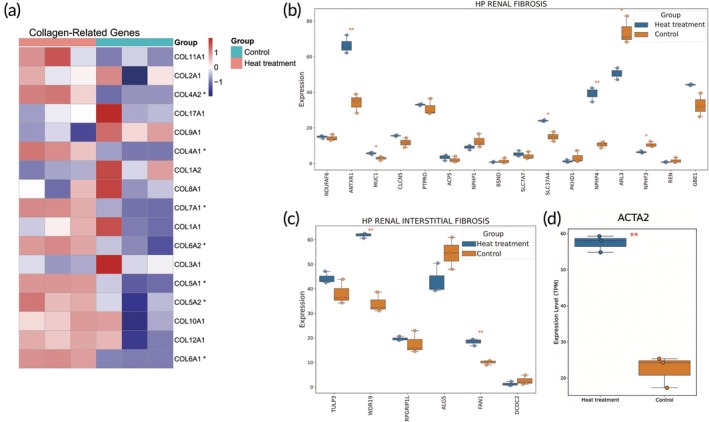
Heat stress induces transcriptional changes associated with fibrosis in kidney organoids. (a) Significant upregulation in the expression of various collagen‐coding genes. (b) Differential gene expression associated with the fibrosis‐related Human Phenotype Ontology term RENAL_FIBROSIS. (c) Differential gene expression associated with the fibrosis‐related Human Phenotype Ontology term RENAL_INTERSTITIAL_FIBROSIS. (d) Significant upregulation of the myofibroblast marker *ACTA2* . *p < 0.05, **p < 0.01, ***p < 0.001, *n* = 3 biological replicates.

Among the collagen‐related genes, *COL4A1*, *COL4A2*, *COL5A1*, *COL5A2*, *COL6A1*, *COL6A2*, and *COL7A1* showed significant upregulation (Figure 6a), suggesting that heat treatment may promote collagen deposition by upregulating these genes. In the context of renal fibrosis phenotype ontology (RENAL_FIBROSIS), upregulation was observed in *ANTXR1*, *MUC1*, *SLC37A4*, and *NPHP4*, while *ARL3* and *NPHP3* were significantly downregulated (Figure 6b). In relation to renal interstitial fibrosis phenotype ontology (RENAL_INTERSTITIAL_FIBROSIS), *WDR19* and *FAN1* exhibited significant upregulation (Figure 6c). Additionally, the myofibroblast marker gene *ACTA2* was notably upregulated, consistent with the fibrotic phenotype observed in Masson staining (Figure 6d).

Our findings demonstrate that heat stress induces significant fibrotic changes in kidney organoids, as evidenced by increased collagen accumulation observed through Masson staining and the substantial upregulation of collagen‐related genes (e.g., *COL4A1*, *COL4A2*, etc.) at the RNA level. Additionally, we observed significant modifications in the expression of fibrosis‐related genes, including *ANTXR1*, *MUC1*, *WDR19*, and *FAN1*. Particularly, the elevated expression of *ACTA2* indicates a potential pivotal role of myofibroblast activation in driving heat‐induced renal fibrosis.

### 3.7 Heat stress induces significant alterations in miRNA, snoRNA, and histone expression

**FIGURE 7:**
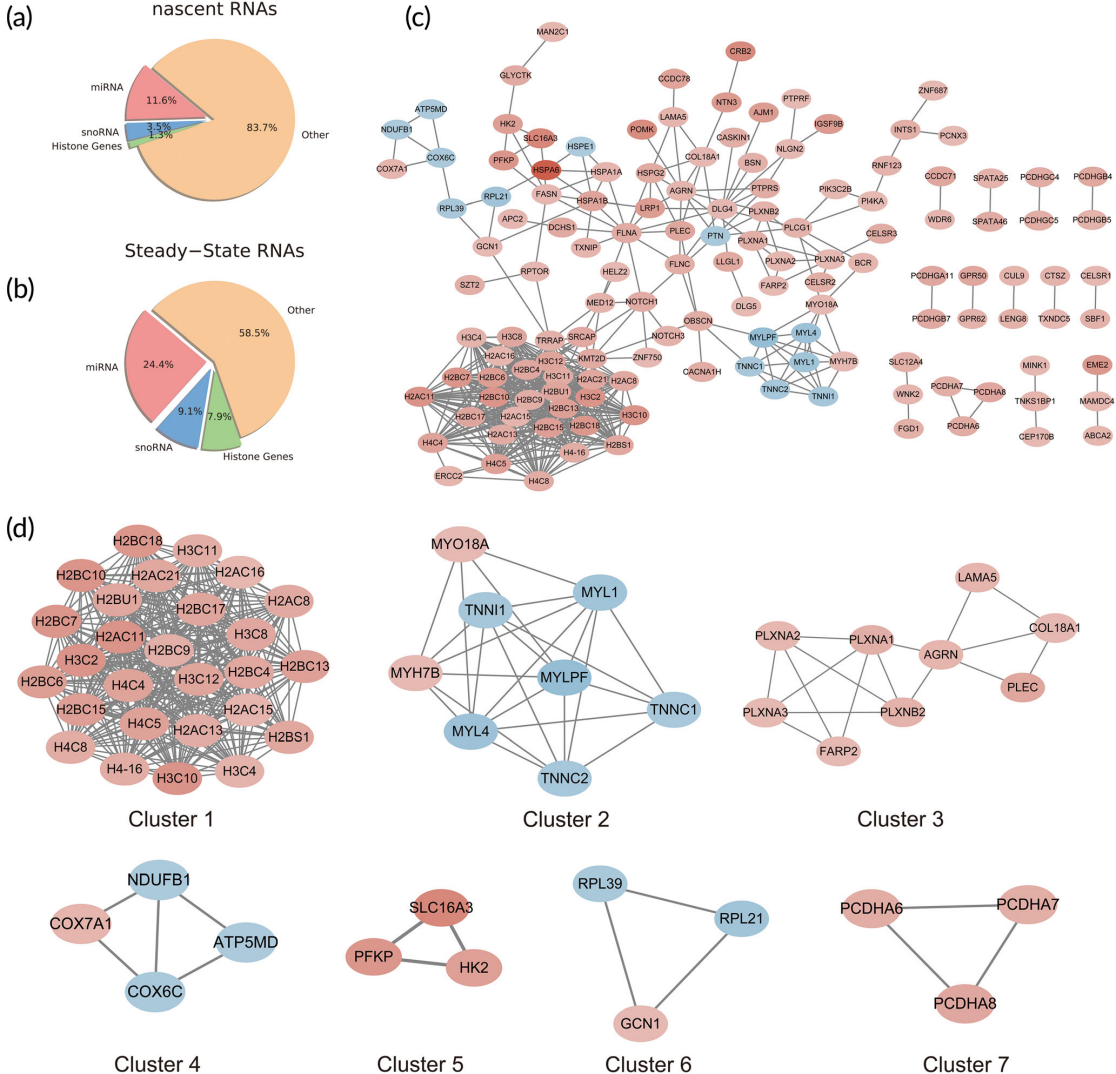
The distribution of miRNA, snoRNA, and histones in differential RNAs and the protein interaction network. (a) The proportion of miRNA, snoRNA, and histones in differential nascent RNAs and (b) steady‐state RNAs revealed a significantly higher proportion in differentially expressed steady‐state RNAs. (c) A protein–protein interaction (PPI) network was constructed using STRING and visualized in Cytoscape. (d) Seven gene modules were identified within the network using MCODE.

An analysis was conducted to evaluate the proportions of miRNA, snoRNA, and histones in differentially expressed nascent and steady‐state RNAs. Our findings revealed that miRNA, snoRNA, and histones accounted for a significantly higher proportion in the differentially expressed steady‐state RNAs compared to nascent RNAs (Figure 7a,b), suggesting greater stability of these molecules during heat stress, enabling them to endure more effectively under stress conditions. Previous research has demonstrated that miRNA, snoRNA, and histones play critical roles in the pathophysiology of AKI.^29^, ^30^, ^31^ Our observations in kidney organoids align well with these findings, further supporting the hypothesis that these molecules contribute to cellular responses and resilience during injury and stress. These results not only provide additional evidence for their involvement in kidney damage but also highlight their potential as biomarkers or therapeutic targets in heat‐induced stress conditions.

A protein interaction network was constructed using STRING and Cytoscape, containing 136 interacting proteins and 537 interaction relationships (Figure 7c and Table S7). Module analysis of the network using MCODE identified seven modules (Figure 7d). The functional annotations of genes in each cluster are presented in Table S8. Cluster 1, composed entirely of histone components, suggests the central role of histones in AKI. Cluster 2 includes genes primarily associated with the cytoskeleton and actin, consistent with F‐actin staining results indicating cytoskeletal damage in kidney organoids post heat treatment. Cluster 3 likely involves the basement membrane, extracellular matrix (ECM) structural components, and extracellular vesicles. Cluster 4 genes are involved in the electron transport chain and cellular aerobic respiration, while Cluster 5 is associated with lactic acid metabolism and glycolysis. Additionally, Cluster 6 is associated with ribosomes and protein translation, and Cluster 7 is related to cell–cell adhesion. These clusters contain key genes responsible for ATP metabolism, potentially influencing the decrease in ATP levels, particularly those that inhibit oxidative respiration and enhance glycolysis. Therefore, we believe that changes in these two clusters could account for the notable decline in ATP levels.

### 3.8 Validation of heat‐induced upregulation in fibrotic‐related biomarkers and hypoxia‐related genes by immunohistochemistry and qPCR

**FIGURE 8:**
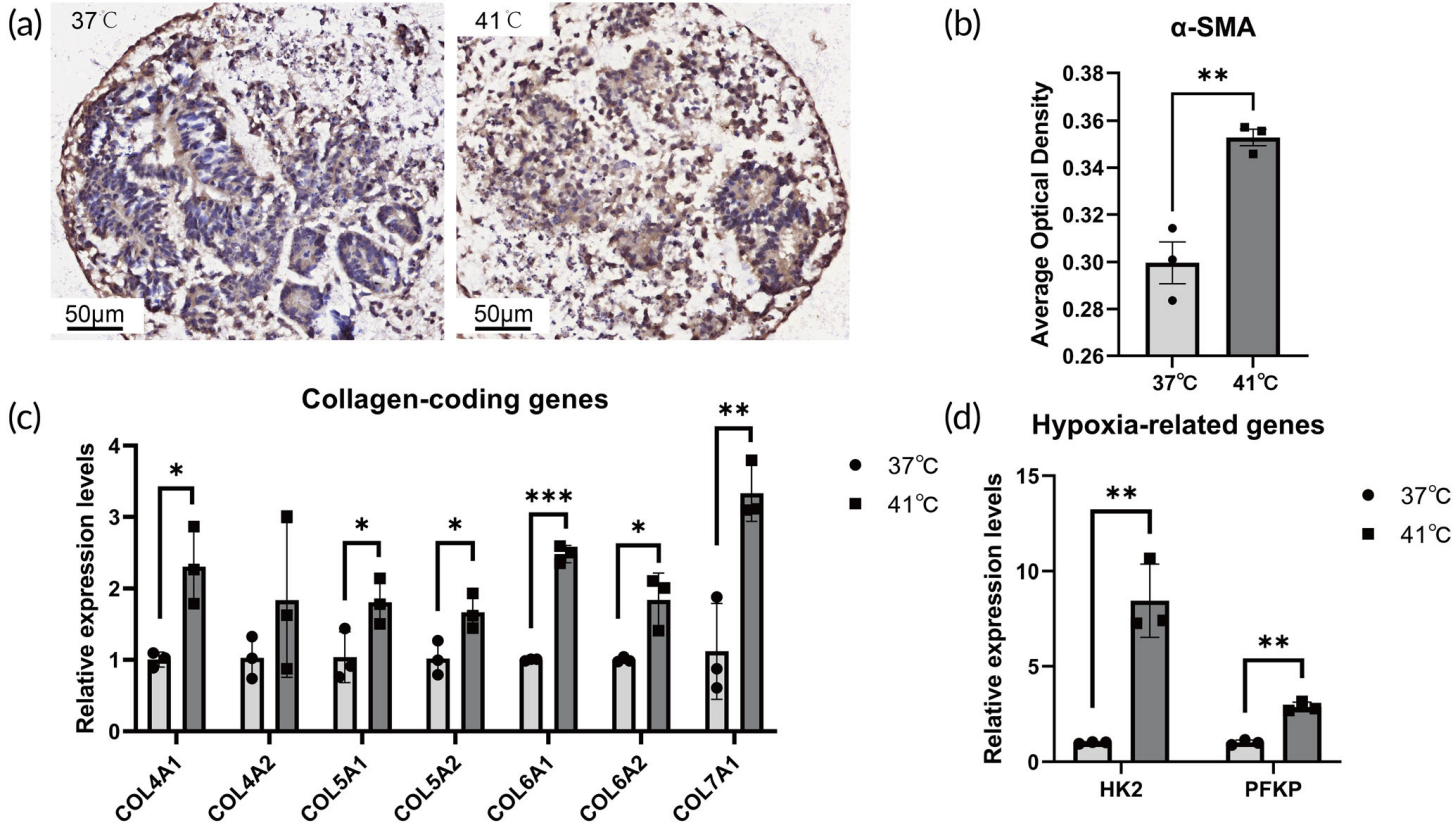
Heat stress induces α‐SMA expression and upregulation of fibrosis‐ and hypoxia‐related genes in kidney organoids. (a) Immunohistochemical staining for α‐smooth muscle actin (α‐SMA) in kidney organoids cultured at 37°C or 41°C for 24 h. Brown staining indicates α‐SMA‐positive cells, while blue nuclei are counterstained with hematoxylin. (b) Quantification of α‐SMA expression via average optical density (AOD) analysis. Data are presented as mean ± SD. (c) qRT‐PCR analysis of collagen‐coding genes (*COL4A1*, *COL4A2*, *COL5A1*, *COL5A2*, *COL6A1*, *COL6A2*, *COL7A1*) in heat‐treated (41°C) vs. control (37°C) kidney organoids. Relative expression levels were normalized to β‐Actin. (d) qPCR validation of hypoxia‐related genes (*HK2*, *PFKP*) in heat‐treated vs. control organoids. Relative expression levels were normalized to β‐Actin. *p < 0.05, **p < 0.01, ***p < 0.001, *n* = 3 biological replicates.

To further validate the fibrotic alterations induced by heat stress, we performed immunohistochemistry staining for α‐SMA and qRT‐PCR for collagen‐encoding genes as well as hypoxia‐related factors. Immunohistochemistry analysis confirmed a significant increase in ɑ‐SMA expression in the 41°C treatment group compared to the 37°C control group, indicating enhanced myofibroblast activation and progressive fibrotic transformation (Figure 8a,b). qRT‐PCR results demonstrated that the mRNA levels of collagen genes *COL4A1*, *COL5A1*, *COL5A2*, *COL6A1*, *COL6A2*, and *COL7A1* were markedly upregulated in heat‐treated organoids, consistent with RNA sequencing data (Figure 8c).

Additionally, qRT‐PCR analysis revealed a significant increase in the expression of *HK2* and *PFKP*, key genes involved in the hypoxia response, underscoring the metabolic reprogramming that contributes to renal fibrosis under heat stress (Figure 8d). These findings collectively reinforce the role of heat stress in promoting ECM deposition and pathological fibrosis in kidney organoids.

## 4 DISCUSSION

This study subjected human iPSCs‐induced kidney organoids to high temperature induced damage, leading to progressive structural damage, observed through optical microscopy. H&E staining indicated apoptosis and necrosis in renal tubules and glomeruli, worsening with prolonged high temperature treatment. Live/dead staining visually confirmed extensive cell death within the kidney organoids after 24 h of high‐temperature exposure. Besides cell necrosis and loss of tubular structures, the absorptive function of the renal tubules was notably impaired. The heat‐stressed organoids exhibited a significant upregulation of four kidney injury biomarkers: IGFBP7, TIMP2, NGAL, and KIM‐1. Especially, urine samples were more effective in detecting these markers compared to serum samples. In conclusion, the results demonstrate the successful establishment of an in vitro model for heat‐stress‐induced AKI.

Mitochondria are essential for the vitality and function of eukaryotic cells, particularly in renal tubular epithelial cells with high energy demands relying on mitochondrial energy supply.^32^, ^33^ The transport function in the proximal tubules requires a high turnover of ATP obtained through mitochondrial oxidative phosphorylation.^34^ In this study, following high‐temperature exposure, a significant decrease in ATP levels was observed in kidney organoids, leading to a corresponding decline in reabsorption function. This reduction in ATP levels may have contributed to the observed damage to the cytoskeletal structure of the kidney organoids, potentially affecting filamentous actin synthesis.^35^

In the PPI network, we identified gene modules associated with AKI. Notably, Cluster 4 and Cluster 5 exhibit gene expression patterns functionally analogous to hypoxic adaptation, known for its significant involvement in oxidative respiration and the electron transport chain. In Cluster 4, *ATP5MD* is part of the proton‐transporting ATP synthase complex.^36^ While *COX7A1* and *COX6C* are components of cytochrome c oxidase, the last enzyme in the mitochondrial electron transport chain driving oxidative phosphorylation.^37^ The downregulation of *ATP5MD* and *COX6C* in Cluster 4 suggests a potential inhibition of oxidative respiration in kidney organoids. Cluster 5 includes three upregulated genes, *HK2*, *PFKP*, and *SLC16A3*, which are crucial in glycolysis and lactate metabolism.^38^, ^39^, ^40^ The differential expression in Cluster 4 and Cluster 5 indicates a reduction in oxidative respiration and an enhancement of the glycolytic pathway in kidney organoids after heat stress. Although glycolysis produces ATP faster than oxidative respiration, it is less efficient, potentially explaining the notable decrease in ATP levels post heat treatment.

Interstitial fibrosis is a definitive pathological change caused by heat‐induced AKI. In high temperature environments, the body is prone to dehydration and heat stress, resulting in insufficient renal perfusion and hypoxia, which subsequently cause damage and apoptosis of renal tubular epithelial cells.^41^ The injured tubular cells release pro‐inflammatory factors, activating inflammatory responses and macrophage infiltration, thereby exacerbating the local inflammatory microenvironment.^42^ Meanwhile, hypoxic conditions stimulate the epithelial‐to‐mesenchymal transition of renal tubular epithelial cells, promoting fibroblast activation and excessive deposition of collagen and ECM. This is one of the key driving factors for the development of renal fibrosis.^43^ Ultimately, these processes collectively contribute to abnormal structural remodeling of the kidney and progressive decline in renal function, leading to irreversible renal fibrosis. In the heat‐treated kidney organoids injury model, Masson staining revealed a significant increase in collagen fiber deposition after exposure to 41°C for 24 h, consistent with the upregulation of collagen‐related genes observed in nascent and stable RNA. It is worth noting that the metabolic reprogramming mediated by *HK2* and *PFKP* in Cluster 5 of the PPI network has been shown in other studies to promote renal interstitial fibrosis.^44^, ^45^ *SLC16A3* is significantly elevated in mouse models of post‐ischemic kidney injury and in severe AKI cases. Inhibiting the protein encoded by *SLC16A3*, monocarboxylate transporter 4 (MCT4), significantly reduces tubular injury and fibrosis.^40^

RNA‐seq analysis of kidney organoids post 24 h heat stress at 41°C revealed no significant upregulation of *COL1A1* or *COL1A2*, the genes encoding type I collagen, a key component of the fibrotic matrix in chronic kidney disease. This absence may be attributed to the acute nature of the injury, preceding the chronic phase where type I collagen is predominant. These observations align with the established model indicating that substantial type I collagen accumulation occurs later in fibrotic progression.^46^ These findings underscore the intricate relationship between metabolic reprogramming and fibrotic pathways in kidney injury, offering insights into potential therapeutic targets for fibrosis mitigation and improved renal outcomes.

RNA‐seq of nascent and steady‐state RNAs was employed to investigate the mechanisms underlying heat‐stress‐induced AKI. GSEA of steady‐state RNAs revealed enrichment of GO, KEGG, and Reactome terms associated with ATP synthesis and energy metabolism. Notably, terms such as oxidative phosphorylation, cytoplasmic translation, ATP synthesis coupled electron transport, mitochondrial ATP synthesis coupled electron transport, aerobic respiration, and the aerobic electron transport chain exhibited significant downregulation. This finding is consistent with our ATP assay conducted on heat‐treated organoids. Mitochondrial dysfunction, leading to impaired biological processes such as oxidative phosphorylation, energy metabolism, and ATP synthesis, has been frequently reported in various types of AKI.^47^, ^48^, ^49^ Our results further demonstrate a significant downregulation of these processes in heat‐induced AKI.

Cluster 1 in the PPI network is predominantly composed of histones, which recent studies have identified as damage‐associated molecular patterns (DAMPs). Necrosis of renal tubular epithelial cells can lead to the release of DAMPs, inducing neutrophils to extrude DNA, histones, and other proteins to form neutrophil extracellular traps (NETs).^50^, ^51^ These extracellular histones can damage the integrity of glomerular endothelial cells, induce further injury to renal tubular epithelial cells, and enhance the formation of NETs.^52^ In our organoid model, the absence of immune cells prevented the verification of NETs formation. Even more unexpectedly, our findings suggest that the elevated histone levels in AKI may not solely be attributed to post‐damage leakage, but could also involve a complex mechanism that triggers an upregulation of histone expression at the transcriptional level.

In this study, we observed numerous differentially expressed genes corresponding to primary miRNAs (pri‐miRNAs) and snoRNAs. Recent evidence indicates that various miRNAs are dysregulated in AKI and contribute functionally to its pathogenesis.^53^, ^54^, ^55^, ^56^ While the biological roles of pri‐miRNAs remain intricate and inadequately characterized, besides serving as precursors for miRNA biogenesis, exhibiting structural similarities to mRNAs and encoding peptides.^57^, ^58^ In addition, snoRNAs are traditionally known to guide RNA modifications and play essential roles in the maturation of other non‐coding RNAs, such as ribosomal RNAs.^59^ Moreover, certain snoRNAs may regulate gene expression in a manner analogous to miRNAs.^60^ For example, in the context of AKI, *SNORD3A* has been reported to modulate STING transcription and promote ferroptosis.^31^ Our findings suggest a potential involvement of pri‐miRNAs and snoRNAs in AKI or renal fibrosis that requires further exploration.

Though kidney organoids have emerged as a valuable tool for modeling kidney diseases and screening potential therapeutics, this technology is constrained by various limitations. Currently available human kidney organoids are still immature, exhibiting molecular and phenotypic characteristics resembling fetal‐like kidney cells and tissues.^19^, ^21^, ^22^, ^61^, ^62^ Moreover, non‐renal off‐target cell populations were found in kidney organoids.^61^ The resulting tissues derived from human iPSCs often lack proper patterning or higher‐order structures^63^, ^64^ and vascularization,^17^, ^19^ all of which are crucial factors for investigating heat‐induced kidney injury and other human kidney diseases.

Despite facing limitations, 3D kidney organoids provide a human‐specific, organotypic‐level in vitro model for research. Through exposure to elevated temperatures, we were able to track alterations in activity and functional integrity of the kidney organoids, as well as assess the expression profiles of clinical biomarkers that are indicative of AKI. This approach enables a comprehensive examination of cellular and molecular responses to heat stress in a controlled, in vitro setting, offering high reproducibility and experimental efficiency while reducing reliance on animal testing. The model facilitates evaluation and development of new therapeutic strategies for heat‐stress‐induced kidney damage. Future research could enhance the model by incorporating immune cells and vascular endothelial cells to improve its physiological relevance; such attempts are in progress.^64^, ^65^

## 5 CONCLUSIONS

This study explored the structural and functional damage of human kidney organoids under heat stress conditions, demonstrating its potential as a model to mimic heat‐stress‐induced AKI. Through RNA‐seq of nascent RNA and steady‐state RNA, significant changes in the organoid's transcriptome were identified, which allow us to better understand the physiological and pathological responses of the kidney to high temperature. By elucidating the underlying mechanisms and pathways linking heat stress to kidney damage, this research provides valuable information for the development of preventive and therapeutic strategies for heat‐induced kidney diseases.

## AUTHOR CONTRIBUTIONS

**Qisheng Su**: Writing – original draft; visualization; software; data curation; methodology; formal analysis; validation. **Liang Yue**: Methodology; validation; visualization; formal analysis; writing – review and editing; data curation; software. **Leixing Ge**: Methodology; validation; formal analysis; software; visualization; supervision; investigation; data curation. **Meida Xiang**: Software; formal analysis; visualization; methodology; investigation; writing – original draft. **Qi Liu**: Methodology; validation; visualization; data curation. **Jiru Wang**: Resources; validation. **Zhimin Yun**: Visualization; formal analysis; software; validation; data curation. **He Liu**: Validation; visualization. **Congji Shan**: Data curation; validation. **Hebing Chen**: Conceptualization; methodology; resources. **Chengjun Wu**: Conceptualization; investigation; writing – review and editing; project administration; resources. **Zhuo Gao**: Conceptualization; investigation; project administration; resources; writing – review and editing. **Yingxia Tan**: Investigation; conceptualization; writing – review and editing; methodology; project administration; resources. All authors have read and agreed to the published version of the manuscript.

## CONFLICT OF INTEREST STATEMENT

The authors declare no conflicts of interest.

## Supporting information


**Figure S1.** Transcriptional profiling reveals kidney organoids exhibiting a fetal‐like maturation stage. Single‐cell RNA sequencing analysis demonstrates robust expression of fetal‐stage patterning genes across nephron domains. The anterior intermediate mesoderm (IM) is characterized by prominent levels of embryonic kidney progenitor markers *LHX1* and *GATA3*, whereas the posterior IM shows elevated expression of *HOXD11*, *EYA1*, and *TBX6*. The specificity of segment maturation is confirmed by the enriched presence of *UMOD* (in the Loop of Henle), *NPHS1* (in the Glomerulus), and *CUBN* (in the Proximal Tubule). Notably, adult kidney markers like *CDH1* exhibit consistently low expression levels across all compartments (*n* = 3 biological replicates). These data indicate a fetal developmental stage of the organoids.

**Figure S2.** Comparative analysis of AKI biomarker expression in nascent vs. steady‐state RNA under heat stress. To elucidate the dynamics of heat‐induced AKI biomarker expression, TPM (transcripts per million) levels of four key biomarkers (*IGFBP7*, *HAVCR1*, *LCN2*, and *TIMP2*) in both nascent and steady‐state RNA fractions were analyzed (*n* = 3 biological replicates).

**Table S1.** Day 0, Day 2, and Day 3 medium.

**Table S2.** Differential_genes of nascent RNAs.

**Table S3.** Differential_genes of steady‐state RNAs.

**Table S4.** GSEA of nascent RNAs.

**Table S5.** GSEA of steady‐state RNAs.

**Table S6.** Transcription_Rate_Ratio_Analysis.

**Table S7.** Protein–protein interaction.

**Table S8.** Function_annotations of genes in MCODE module.

## Data Availability

The data that support the findings of this study are available from the corresponding author upon reasonable request.
