# Supplementary figures and images for: Kidney organoids as a novel platform to evaluate heat‐stress‐induced acute kidney injury pathogenesis
Source: Bioeng Transl Med. 2025 Nov 20;11(1):e70092. doi: 10.1002/btm2.70092 (PMC12821206; doi:10.1002/btm2.70092)

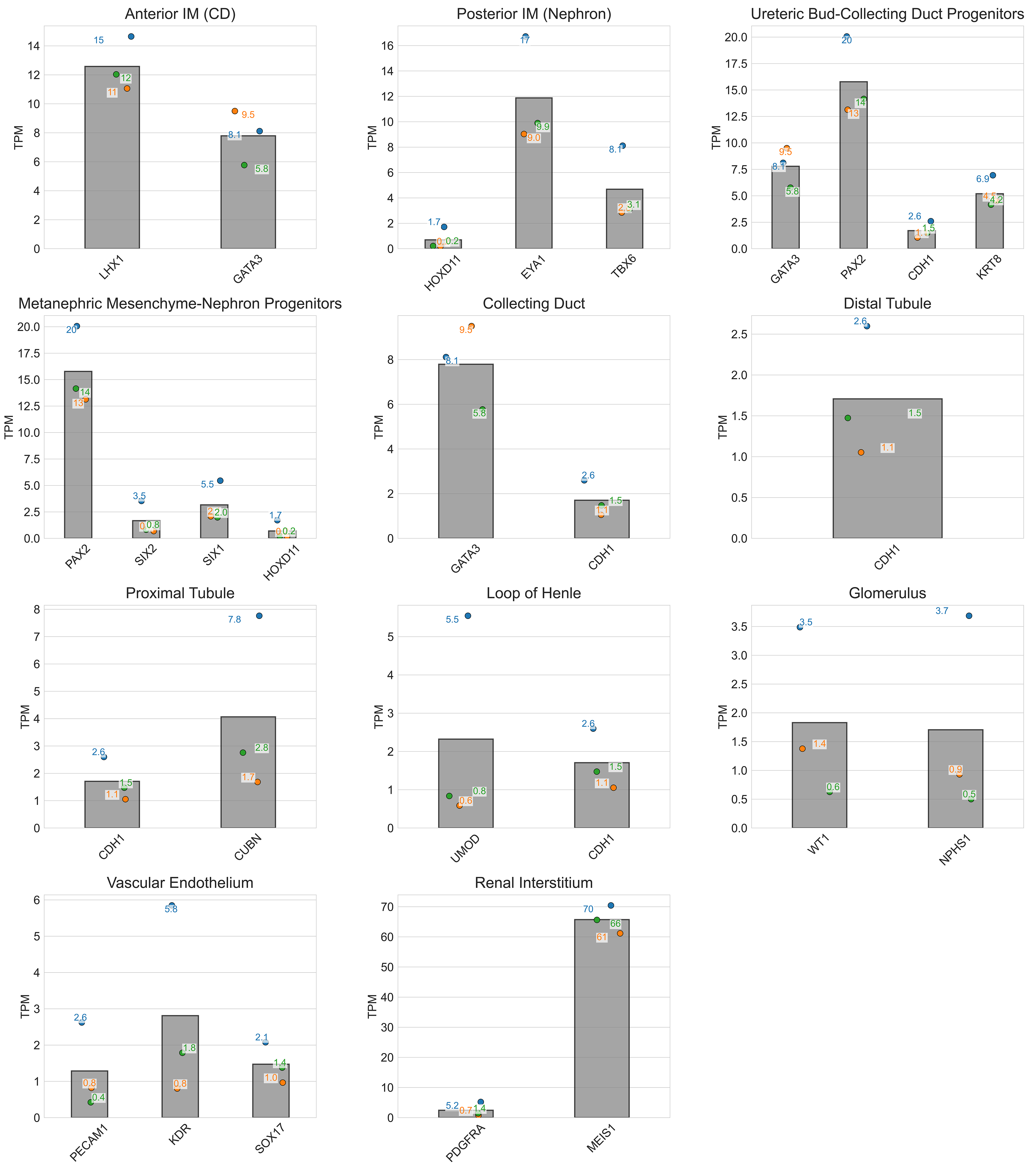

Supplement: Supplementary file 1 — Figure S1. Transcriptional profiling reveals kidney organoids exhibiting a fetal‐like maturation stage. Single‐cell RNA sequencing analysis demonstrates robust expression of fetal‐stage patterning genes across nephron domains. The anterior intermediate mesoderm (IM) is characterized by prominent levels of embryonic kidney progenitor markers LHX1 and GATA3, whereas the posterior IM shows elevated expression of HOXD11, EYA1, and TBX6. The specificity of segment maturation is confirmed by the enriched presence of UMOD (in the Loop of Henle), NPHS1 (in the Glomerulus), and CUBN (in the Proximal Tubule). Notably, adult kidney markers like CDH1 exhibit consistently low expression levels across all compartments (n = 3 biological replicates). These data indicate a fetal developmental stage of the organoids. [file BTM2-11-e70092-s001.tif]
